# Evolving dynamics of insect frass fertilizer for sustainable nematode management and potato production

**DOI:** 10.3389/fpls.2024.1343038

**Published:** 2024-02-23

**Authors:** Emmanuel O. Anedo, Dennis Beesigamukama, Benson Mochoge, Nicholas K. Korir, Solveig Haukeland, Xavier Cheseto, Sevgan Subramanian, Segenet Kelemu, Chrysantus M. Tanga

**Affiliations:** ^1^ International Centre of Insect Physiology and Ecology, Nairobi, Kenya; ^2^ Department of Agricultural Science and Technology, Kenyatta University, Nairobi, Kenya; ^3^ Crop Research Operations Department, National Root Crops Research Institute, Umudike, Abia State, Nigeria; ^4^ Division of Biotechnology and Plant Health, Norwegian Institute for Bioeconomy Research (NIBIO), Ås, Norway

**Keywords:** potato cyst nematodes, chitin, black soldier fly frass fertilizer, regenerative soil input, soil health, potato yield

## Abstract

Potato production faces major challenges from inadequate soil fertility, and nematode infestation, yet synthetic fertilizers and nematicides are costly and harmful to the environment. This study explored the potential of chitin-fortified black soldier fly-composted organic fertilizer (BSFCOF) as a multipurpose organic fertilizer amendment for enhancing potato yield and suppressing potato cyst nematodes (PCN). The BSFCOF was applied at a rate equivalent to 150 kg N ha^-1^ and fortified with chitin from black soldier fly pupal exuviae at inclusion rates equivalent to 0.5, 1, 2, 3, 4 and 5% chitin. Data were collected on potato growth characteristics, PCN population densities, and soil chemical properties for two growing cycles. Results showed that chitin fortified BSFCOF significantly improved potato growth parameters, chlorophyll concentration, marketable tuber yield and number of marketable tubers. The marketable tuber yield achieved using chitin-fortified BSFCOF was 70 – 362%, and 69 – 238% higher than the values achieved using unfertilized soil during the first and second growing cycles, respectively. Soil amendment with chitin-fortified BSFCOF significantly reduced the number of cysts per 200 g soil^-1^, number of eggs and J2 per cyst^-1^, eggs g^-1^ soil and reproduction rate by 32 – 87%, 9 – 92%, 31– 98% and 31 – 98%, respectively. The PCN suppression increased with chitin inclusion rates. There were significantly higher values for soil pH, ammonium nitrogen, nitrate nitrogen, available phosphorus, calcium, magnesium, potassium, and cation exchange capacity in soil amended with BSFCOF compared to unamended soil. This study demonstrates that BSFCOF fortified with 5% chitin is an effective soil enhancer with multiple benefits, including improved soil fertility, potato performance, and effective management of potato cyst nematodes.

## 1 Introduction

Crop production in sub-Saharan Africa (SSA) and most developing countries is at risk due to soil degradation, climate change, pest invasion among others (Tully et al., 2015; Montanarella et al., 2016; Constantine et al., 2020; FAO et al., 2023). Inadequate soil and nutrient management practices often lead to nutrient deficiencies, erosion, and biodiversity losses in many farmlands across SSA (Tully et al., 2015; Raimi et al., 2017; Wortmann et al., 2019). Smallholder farmers in SSA apply insufficient amounts of mineral fertilizers which in most cases do not meet crop nutrient demands (Adewumi and Adebayo, 2008; Bergh et al., 2012; FAO, 2017), due to high fertilizer purchase costs, inaccessibility, and reliance on fertilizer imports (Yeng et al., 2012; Liverpool-Tasie et al., 2015; WFP (World food programme), 2022). Exclusive application of mineral fertilizers has been associated with soil acidification, soil nutrient imbalances, greenhouse gas emissions, and failure to restore soil organic matter that is essential for achieving optimal crop yields (Raimi et al., 2017).

Extensive soil degradation, intensive agriculture and climate change is likely to increase the proliferation of soil-dwelling pests especially plant parasitic nematodes (Coyne et al., 2018; Dutta and Phani, 2023). For example, the emergence of potato cyst nematodes (PCN) (*Globodera rostochiensis* and *Globodera pallida*) in Kenya and other sub-Saharan African countries poses a significant threat to potato productivity, with an estimated 80% reduction in yield (Singh et al., 2013; Mwangi et al., 2015; Mburu et al., 2018, 2020). Despite being recognized as one of the most important quarantine pests, PCNs are usually overlooked in Africa and other developing countries (Coyne et al., 2018; Niere and Karuri, 2018). Globally, plant parasitic nematodes cause significant crop losses, with root-knot and potato cyst nematodes contributing to an annual yield loss of about 12.3% and 9%, respectively, and causing an estimated monetary loss of over US$ 118 billion (McCarter, 2009; Turner and Subbotin, 2013; Jang et al., 2014). While chemical nematicides are the most commonly used method for nematode control, their prohibitive cost to smallholder farmers and negative effects on non-target organisms and human health have led to the pursuit for sustainable alternatives (Chitwood, 2003; Zhang et al., 2017; Sabarwal et al., 2018; Touray et al., 2021).

The combined challenges of soil fertility depletion and nematode infestation have caused significant reduction in the yields of key food crops such as potatoes. In Kenya, potato is the second most consumed staple food crop after maize, and potato production provides livelihoods for over 800,000 Kenyans, benefiting over 2 million others in the production chain (CIP, 2019 ; Abong and Kabira, 2013). However, the average potato yield in the major potato growing areas of Kenya is low at 8 – 15 t ha^-1^, compared to the global yield potential of 20 – 40 t ha^-1^ (Muthoni and Nyamongo, 2009; Kiptoo et al., 2016; Muthoni, 2016; VIB, 2019). This is mainly due to poor soil fertility, unbalanced nutrient application, poor crop rotation practices, soil erosion, biodiversity loss, high mineral fertilizer prices and nematode infestation among other factors (Bationo, 2004; Muthoni and Nyamongo, 2009; Muthoni, 2016; Mburu et al., 2020; Mugo et al., 2020). The use of organic fertilizers for enhancing soil quality, boosting crop productivity, and pest control is gaining worldwide recognition (FAO et al., 2023), primarily due to the high prices of mineral fertilizers, as well as their adverse impacts human health and the environment.

The organic fertilizer obtained from mass rearing of black soldier fly (*Hermertia illucens* L.) for animal feed is gaining global recognition as an eco-friendly substitute for mineral fertilizers and synthetic pesticides in crop production (Beesigamukama et al., 2023a, Beesigamukama et al., 2023b). Black soldier fly-composted organic fertilizer (BSFCOF) and chitin-rich exuviae contain essential nutrients which are readily available to crops (Beesigamukama et al., 2020a, Beesigamukama et al., 2021; Menino et al., 2021). Recent studies have demonstrated that these by-products possess the potential to enhance the activities of beneficial soil microorganisms (Barragán-Fonseca et al., 2022; Wantulla et al., 2023a), augment plant development and trigger systemic resistance in plants (Quilliam et al., 2020; Barragán-Fonseca et al., 2022), attract parasitoids and plant pollinators, and significantly contribute to the suppression of soil-borne plant pathogens and pests (Temple et al., 2013; Poveda et al., 2019; Quilliam et al., 2020; Wantulla et al., 2023b). In SSA for example, the potential of these by products, especially BSFCOF to improve maize yield (Beesigamukama et al., 2020a, Beesigamukama et al., 2020b; Tanga et al., 2021), kale, tomato, and French bean (Anyega et al., 2021), kale and Swiss chard (Abiya et al., 2022) has been demonstrated, and insect-composted organic fertilizer has been recommended as a complement or total replacement of mineral fertilizer.

Application of chitin or chitin-rich organic amendments have also been found effective in suppressing plant-parasitic nematodes and boosting crop growth and yield (Oka, 2010; Hallmann et al., 1999; Rodriguez-Kabana et al., 1987; Sarathchandra et al., 1996). Soil amendment with crustacean derived chitin reduced the number of Beet cyst nematode (*Heterodera schachtii*) and eggs by 44% and 35%, respectively (Spiegel et al., 1988a). Also, application of synthetic chitin or chitin-enriched compost reduced the galling indices of *Meloidigyne incognita* (Jin et al., 2005) and *M. javanica* (Hussain et al., 2013). Apart from plant parasitic nematodes, chitin or chitin-rich organic amendments are effective in addressing biotic stresses such as bacterial wilt (*Ralstonia solanacearum*) in tomatoes (Kemboi et al., 2022), Root rot in chilli (*Fusarium. solani*) (Hussain et al., 2013), symptoms of *Rhizoctonia. solani* in sugar beet (Andreo-Jimenez et al., 2021), and aphid infestations in cotton (Rajendran et al., 2011).

The increase in the activities of chitinolytic microorganisms (Wantulla et al., 2023a; Spiegel et al., 1987; Rodriguez-Kabana et al., 1987), release of ammonia (Oka, 2010), improved soil chitinase activity (Jung et al., 2002; Jin et al., 2005) and production of antibiotics (Akhtar and Malik, 2000; Ali et al., 2002) have been reported as some of the possible mechanisms behind the biocontrol efficacy of chitin and chitin-rich organic soil amendments in plant parasitic nematode control. Strohl (1997) reported that enriching compost with shrimp shell chitin increased the abundance of Gram-positive bacteria, the group known for having an important role in antibiotic production, while Jin et al. (2005) found that increase in chitinase activity was associated with reduced galling index of *M. incognita*.

Previous studies on the use of chitin in pest and disease management primarily used synthetic chitin, which is expensive and limited in availability though chitin is the second most abundant polysaccharide. Therefore, the prohibitive costs and limited access associated with synthetic chitin have hindered its wide application for the control of soil-borne pests such as nematodes. Realizing the full potential of chitin in the control of nematodes requires strategies that can avail sustainable, affordable and accessible sources for smallholders. In Africa, there are more than 2,300 insect-based enterprises dealing in different products including frass fertilizer, insect chitin, insect-based feeds, foods and oils, highlighting the immense business potential of this emerging industry (Tanga and Kababu, 2023). Black soldier fly pupal exuviae contains 10 – 30% chitin depending on the extraction method (Hahn et al., 2020; Lagat et al., 2021). Insect mass production systems such as those of black soldier fly produce huge amounts of pupal shells, also known as exuviae that are rich in chitin (Lagat et al., 2021) and could be exploited for nematode control. This can be achieved by applying the exuviae alone or in combination with insect frass fertilizer to produce a multipurpose organic amendment that can supply nutrients and suppress soil-borne pests and pathogens (Kemboi et al., 2022). For example, by adding chitin-rich exuviae from BSF pupae to the composted organic frass fertilizer, it may be possible to create a fertilizer that not only provides essential plant nutrients but also enhances the soil’s ability to suppress potato cyst nematodes. However, this is an emerging research area, and knowledge on the appropriate inclusion rates of BSF pupal exuviae into the frass fertilizer for optimal plant growth, higher potato yield, and effective control of potato cyst nematodes is scant. Therefore, this study seeks to investigate the efficacy of chitin-rich BSF pupal exuviae combined with composted frass fertilizer on potato growth and yield, PCN control and soil chemical properties, to generate information necessary for their use as cost-effective and regenerative inputs for PCN management potato production.

## 2 Materials and methods

### 2.1 Study location and source of materials

The study was carried out at the International Centre of Insect Physiology and Ecology (*icipe*), Nairobi, Kenya (1°13’27.3”S 36°53’48.5”E) between April 2022 and February 2023. The black soldier fly-based chitin was obtained from BSF pupal exuviae sourced from a BSF colony maintained at *icipe*’s Duduville campus. The BSF pupal exuviae (pupal casings) were washed using tap water, sun dried for 4 days, and ground into a fine powder (< 2mm) using KM-400 mechanical grinder (MRC laboratory equipment and manufacturing UK). The BSFCOF was obtained by feeding the black soldier fly larvae on brewer’s spent grain (barley waste) according to the procedure outlined by Shumo et al. (2019). The larvae were harvested after two weeks, while the frass was composted for 5 weeks using the heap method as described by Beesigamukama et al. (2020a). The soil used for the experiment was sourced from *icipe* experimental farm, sieved (2 mm sieve) to remove debris and autoclaved for 40 minutes at 121°C to eliminate any PCN present.

### 2.2 Treatments and experimental setup

The treatments comprised of BSFCOF applied at a rate equivalent to 150 kg N ha^-1^ (Otieno and Mageto, 2021). The BSFCOF was amended with BSF pupal exuviae powder at inclusion rates equivalent to 0, 0.5, 1, 2, 3, 4, and 5% chitin (weight/weight). The pupal exuviae contains 10.2‐11.9% chitin depending on the extraction method (Lagat et al., 2021). The control treatment consisted of unamended soil. The treatments were denoted as BSFCOF (supplies 100% N), BSFCOF+0.5% chitin (100% N+0.5% exuviae), BSFCOF+1% chitin (100% N+1% exuviae), BSFCOF + 2% chitin (100% N+2% exuviae), BSFCOF+3% chitin (100% N+3% exuviae), BSFCOF+4%chitin (100% N + 4% exuviae), BSFCOF+5% chitin (100% N+5% exuviae) and Control (unamended soil). The experiment was conducted in 10-liter bucket (Kenpoly smiley 10 bucket, Kenpoly Manufacturers Ltd Nairobi, Kenya) containing 9 kg of sterilized soil. The soil was amended with a mixture of BSFCOF and exuviae according to the treatments. The potting mixtures were maintained at 60% field capacity and left for one week before planting. One tuber (25 – 30g) of pre-sprouted and pest-free susceptible potato variety (Cv Shangi) was planted in each pot at a depth of 5cm. The trial was arranged in a randomized complete block design (RCBD) with four replications, and repeated once. The first cycle was conducted between April and August 2022, while the second cycle began in October 2022 and ended in February 2023.

### 2.3 Nematode sampling and inoculum preparation

Soil samples were collected from potato farms with a high incidence of PCN infestation in Nyandarua County, a major potato-producing area in Kenya (Mburu et al., 2020). The collected soil samples were air-dried for 7days. Cysts were extracted using the Fenwick can flotation method from a 200 g sample (EPPO, 2013). The extracted cysts were collected on milk filter paper, air-dried, and manually picked using entomological forceps under a LEICA EZ4 stereomicroscope (Leica Microsystems GmbH. Germany). The viability of the cysts was assessed using Nile blue A (Sigma Aldrich, USA), and 20 cysts of equal size (in triplicates) were selected for assessment (Mburu et al., 2020). After 48 hours of incubation, the cysts were crushed to expose the viable eggs, non-viable eggs and second-stage juveniles (J2). The stained eggs (non-viable), non-stained eggs (viable), and live (viable) second-stage juveniles (J2) were identified using a LEICA M80 stereomicroscope at ×40 magnification (Leica Microsystems GmbH. Germany). The cyst fertility (CF) was determined as the sum total of the number of J2s, stained eggs, and non-stained eggs. Cyst viability (CV%) was determined using Equation 1 (Mburu et al., 2020).


(1) $$\text{Cyst viability }\left(\%\right)=\;\frac{\left(\text{number of J}2\text{s}\right)+\left(\text{number of non}-\text{stained eggs}-\text{stained eggs}\right)}{\text{Cyst fertility }}\times 100\;$$

The number of viable eggs and J2 per cyst was determined by counting under LEICA M80 stereomicroscope at ×40 magnification and the average was found to be 192.7 eggs and J2 per cyst. This was used to estimate the number of cysts to be inoculated per pot, taking the volume of soil and initial nematode population (2 eggs and J2 g soil^-1^) into account. Three weeks after potato emergence, each pot was inoculated with 93 cysts at an initial population density of 2 eggs and J2 g soil^-1^ (Renčo et al., 2016). The eggs and J2s were enclosed in muslin bags to prevent mixing with newly formed cysts, and placed near the rhizosphere of each plant.

### 2.4 Potato growth, yield and nematode populations

Potato leaf growth, plant height, stem diameter, and leaf chlorophyll concentration were determined at 4, 6, 8, 10, and 12 weeks after planting. Plant height was determined using a tape measure that was placed from the soil level to the tip of the plant. Number of leaves were determined by counting the fully-developed and photosynthetically active leaves. Leaf chlorophyll concentration was determined using a chlorophyll meter (SPAD-502plus, Konica Minolita, Japan) that was placed on 6 fully developed leaves from the top. Stem diameter was measured using a vernier calliper (150 mm, Toolstream Ltd., UK), that was placed at 10 cm from the soil level. At harvesting (i.e., 3 months and 2 weeks after planting), the potato plants were carefully uprooted from each pot to retrieve the tubers. The number of marketable tubers (i.e., those that were pest and diseases free and weighing above 25g) per pot were counted and separated from non-marketable. The tuber weights per pot were measured using an electronic weighing balance, and thereafter converted to tonnes per hectare (t ha^-1^) (Ebrahim et al, 2018; Asnake et al., 2023).

After harvesting, the soil in each pot was thoroughly mixed together and quarter sampling was carried out to obtain a sub-sample of 200g was taken for cyst extraction following procedures described in section 2.3. The number of cysts extracted from each pot was counted and the viability was assessed using Nile blue stain. The effectiveness of the chitin-fortified BSFCOF in PCN suppression was determined by calculating cyst viability (Equation 1) and nematode multiplication rate (Equation 2).


(2) $$\text{Rf}=\;\frac{\text{Pf}}{\text{Pi}}\;$$

Where:

Rf is the reproduction factor.

Pf is the final nematode population and Pi is the initial nematode population (Vanitha et al., 2019).

### 2.5 Soil and fertilizer analysis

The BSFCOF, exuvaie, pre- and post- planting soil samples were analyzed for nutrients and other chemical properties using standard laboratory methods (**Table 1**). Particle size distribution was determined using the Bouyocous hydrometer method and textural class determined using soil textural triangle (Gee and Or, 2002). The pH of both soil and the fertilizer was determined using glass electrode pH meter (AD1000, Adwa, Bucharest, Romania) and while the electrical conductivity was determined using EC meter (AVI, Labtech, Mumbai, India) (Okalebo et al., 2002). Organic carbon was determined using wet oxidation method as described by Nelson and Sommers (1996). Total nitrogen was determined using Kjeldahl digestion and distillation method, while the mineral nitrogen (nitrate and ammonium) was extracted using 0.5 M potassium sulphate at a ratio of 1:10 (w/v) and determined colorimetrically at 419 and 655 nm, respectively (Okalebo et al., 2002). Available P, exchangeable K, and exchangeable Ca, and Mg were extracted using acid digestion (Okalebo et al., 2002). The exchangeable Ca and Mg concentrations in the samples were determined using atomic absorption spectrometry (AAS), at 422.7 and 285.2 nm, respectively (iCE 3300 AA system, Thermo Scientific, Shanghai, China). Available P (Bray 2) and exchangeable K was determined using flame photometry. The cation exchange capacity was determined by leaching the soil sample with 1M NH4OAc at pH 7 (Bremner, 1996). Micronutrients (iron, zinc, copper, boron and manganese were determined using Mehlich I method and analyzed using atomic absorption spectrometry (Mehlich, 1953).

**Table 1:** Chemical properties of the experimental soil, blacksoldier fly-composted organic fertilizer, and the pupal exuviae.

Parameter	Soil	BSFCOF	Pupal exuviae
pH (H_2_O)	6.98	5.49	6.95
Electrical conduxtivity(mS/cm)	–	11.8	4.39
Organic carbon (%)	1.14	43.9	42.0
Nitrogen (%)	0.10	3.69	5.52
Available phosphorus (%)	0.076	1.54	0.49
Exchangeable potassium (%)	0.032	2.37	1.16
Exchangeable calcium (%)	0.14	1.00	4.99
Exchangeable magnessium (%)	0.002	0.59	0.39
Manganesse (ppm)	548	301	2420
Iron (ppm)	136	5710	5300
Zinc (ppm)	13.5	191	114
Copper (ppm)	1.96	31.8	11.7
Boron (ppm)	0.75	26.9	18.8
Exchangeable sodium (ppm)	0.01	3100	2620
Carbon/nitrogen ratio	12.0	11.9	7.61
Cation exchange capacity (Cmol kg^−1^)	10.0	–	–
Acid saturation (%)	0.52	–	–
Exchangeable acidity (meq/100g)	0.05	–	–
Sand (%)	40.2	–	–
Silt (%)	29.1	–	–
Clay (%)	30.7	–	–
Textural class	Clay loam	–	–

BSFCOF, black solder fly-composted organic fertilizer.

### 2.6 Statistical analysis

The normality of the collected data was tested using the Shapiro-Wilk test. A linear mixed-effect model was applied to analyze the data on plant height, number of leaves, stem diameter, and chlorophyll concentration, using the ‘lmer’ function from the ‘lme4’ package. In this model, the fixed effects were the fertilizer treatments and sampling time, while replication was kept as a random effect. The data on the number of marketable tubers, marketable tuber yield (t/ha), and soil chemical properties were analyzed using a one-way analysis of variance test. Least squares means were calculated using the “lsmeans” package, and significant means were separated using the “Tukey” test at a significance level of P ≤ 0.05. The data on growth, yield, soil chemical properties and nematode population parameters were analyzed separately for each cycle. To examine the relationship between soil chemical properties, potato growth, yield, and nematode population densities, principal component analysis (PCA) was performed using “prcomp” function from “ggbiplot” packages. Data for the two cycles were pooled for this analysis. The statistical analyses were carried out using R software (R Core Team, 2022).

## 3 Results

### 3.1 Impact of BSFCOF and chitin-fortified BSFCOF on potato growth

**Figure 1:**
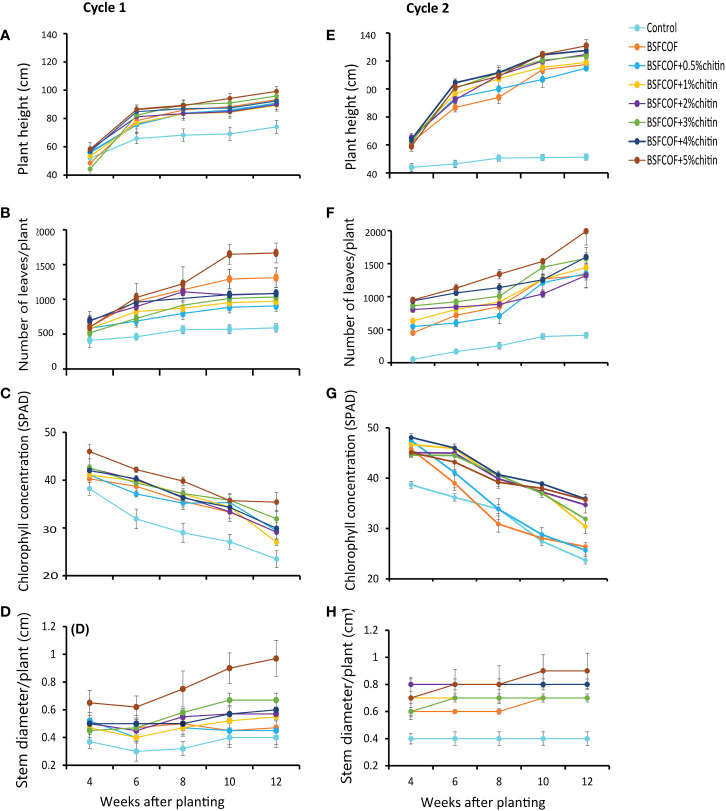
Effect of chitin-fortified black soldier fly-composted organic fertilizer on potato height **(A, E)**, number of leaves, **(B, F)** chlorophll concentrations **(C, G)**, and stem diameter **(D, H)**, during the first **(A–D)** and second **(E–H)** experimental cycles. Control, no fertilizer amendment; BSFCOF, black soldier fly-composted organic fertilizer; BSFCOF+0.5% chitin, black soldier fly-composted organic fertilizer amended with 0.5% BSF pupal chitin; BSFCOF+ 1% chitin, black soldier fly-composted organic fertilizer amended with 1% BSF pupal chitin; BSFCOF+ 2% chitin, black soldier fly-composted organic fertilizer amended with 2% BSF pupal chitin; BSFCOF+ 3% chitin, black soldier fly-composted organic fertilizer amended with 3% BSF pupal chitin; BSFCOF+ 4% chitin, black soldier fly-composted organic fertilizer amended with 4% BSF pupal chitin; BSFCOF+ 5%, chitin black soldier fly-composted organic fertilizer amended with 5% BSF pupal chitin.

The potato height varied significantly due to fertilizer amendments (first cycle: χ2 = 68.6, df =7, *P<* 0.001, second cycle: χ2 = 541.3, df =7, *P*< 0.001) and potato growth stage (first cycle: χ2 = 415.3, df =4, *P*< 0.001, second cycle: χ2 = 561.9, df =4, *P*< 0.001) (**Figures 1A, E**). The interaction effect of fertilizer amendments and growth stage was significant in the second cycle only (χ2 = 105.9, df =28, P< 0.001, first cycle: χ2 = 26.7, df =28, *P* = 0.53). Compared to the control, the fertilizer treatments significantly (*P*< 0.001) increased the plant height by 17 – 25% and 46 – 54% in the first and second cycles, respectively. The potato height followed an increasing trend and reached to peak levels in the 12^th^ week. The plant height increased with an increase in the chitin inclusion. BSFCOF + 5% chitin produced the tallest plants which were 3 – 10% and 3 – 12% higher than the values achieved using other treatments in the first and second cycles, respectively.

The number of leaves was significantly influenced by the fertilizer amendments (first cycle: χ2 = 177.5, df =7, *P*< 0.001, second cycle: χ2 = 510.0, df =7, *P*< 0.001), growth stage (first cycle: χ2 = 151.2, df =4, *P*< 0.001, second cycle: χ2 = 365.5, df =4, *P<* 0.001), and their interactions (first cycle: χ2 = 46.9, df =28, *P*< 0.05, second cycle: χ2 = 43.2, df =28*, P*< 0.05) (**Figures 1B, F**). The fertilizer amendments significantly (*P*< 0.001) increased the number of leaves relative to the control by 35 – 65% and 55 – 61% in the first and second growing cycles, respectively. Leaf growth increased with chitin inclusion rates. Soil amendment with BSFCOF + 5% chitin achieved the highest number of leaves which were 21– 46% and 21– 36% higher than the values achieved using other fertilizer treatments during the first and second growing cycles, respectively.

The potato leaf chlorophyll concentration also varied significantly due to fertilizer amendments (first cycle: χ2 = 150.2, df = 7, *P*< 0.001, second cycle, χ2 = 342.5, df = 7, *P<* 0.001) and growth stage (first cycle: χ2 = 396.3, df = 4, *P*< 0.001, second cycle: χ2 = 860.8, df = 4, *P*< 0.001). The interaction effect was significant in the second cycle (χ2 = 95.8, df = 28, *P<* 0.001), but not the first cycle (χ2 = 21.9, df = 28, *P* = 0.78) (**Figures 1C, G**). In both growing cycles, a decreasing trend in the leaf chlorophyll concentration was observed throughout the experiments. However, the fertilizer amendments increased the chlorophyll concentration by 13 – 34% and 8 – 34% compared to the control during the first and second growing cycles, respectively. Soil amendment with BSFCOF + 5% chitin produced potatoes with the highest leaf chlorophyll concentration at the 4^th^ week, that was 10 – 34% and 0.6 – 26% higher than the values obtained using other treatments in the first and second cycles, respectively.

The potato stem diameter exhibited significant variations due to the fertilizer amendments (first cycle: χ2 = 121.8, df = 7, *P*< 0.001, second cycle, χ2 = 170.9, df = 7, *P*< 0.001) and growth stages (first cycle: χ2 = 23.0, df = 4, P< 0.001, second cycle: χ2 = 11.7, df = 4, *P*< 0.05). The interaction effect was not significant in both cycles (first cycle: χ2 = 22.4, df = 28, *P* = 0.76, second cycle: χ2 = 7.0, df = 28, *P* = 0.999) (**Figures 1D, H**). The fertilizer amendments significantly increased (*P<* 0.001) the stem diameter by 20 – 60% and 42 – 56% compared to the control during the first and second growing cycles respectively. The stem diameter increased in most cases with increase in the chitin inclusion levels. The highest stem diameter was achieved with soil amendment with BSFCOF + 5% chitin, which were 30 – 50% and 11 – 22% higher than the values achieved using other fertilizer treatments during the first and second cycles, respectively.

### 3.2 Influence of BSFCOF and chitin-fortified BSFCOF on potato yield

**Figure 2:**
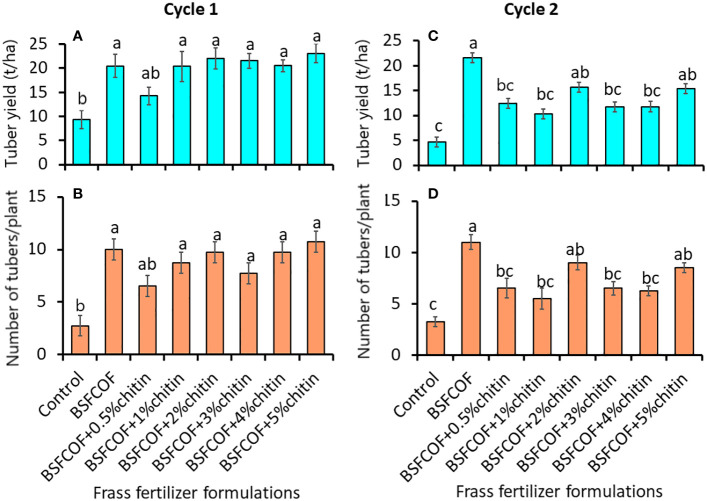
Effect of chitin-fortified black soldier fly-composted organic fertilizer on potato tuber yield **(A, C)** and number of marketable tubers **(B, D)** during the first **(A, B)** and second **(C, D)** experimental cycles. Control, no fertilizer amendment; BSFCOF, black soldier fly-composted organic fertilizer; BSFCOF+0.5% chitin, black soldier fly-composted organic fertilizer amended with 0.5% BSF pupal chitin; BSFCOF+ 1% chitin, black soldier fly-composted organic fertilizer amended with 1% BSF pupal chitin; BSFCOF+ 2% chitin, black soldier fly-composted organic fertilizer amended with 2% BSF pupal chitin; BSFCOF+ 3% chitin, black soldier fly-composted organic fertilizer amended with 3% BSF pupal chitin; BSFCOF+ 4% chitin, black soldier fly-composted organic fertilizer amended with 4% BSF pupal chitin; BSFCOF+ 5%, chitin black soldier fly-composted organic fertilizer amended with 5% BSF pupal chitin. Per panel, mean (± standard error) followed by the same letters are not significantly different at p< 0.05.

The chitin-fortified BSF-organic fertilizer formulations significantly increased both the marketable tuber yield (first cycle: *F*_(7, 24)_ = 5.08, *P*< 0.01, second cycle: *F*_(7, 24)_ = 7.31, *P*< 0.001) and number of marketable potato tubers (first cycle: *F* = 6.56, *P<* 0.001, second cycle: *F* = 8.29, df = 7, 24, *P<* 0.001) (**Figures 2A–D**). The fertilizer amendments significantly (*P*< 0.001) increased the marketable tuber yield by 70 – 148% and 121 – 362% compared to the control during the first and second cycles, respectively. It was noted that unfortified BSFCOF produced 34 – 66% significantly (*P*< 0.001) higher potato tuber yield compared to chitin-fortified BSFCOF treatments during the second cycle, except BSFCOF+2% chitin and BSFCOF+5% chitin.

The number of marketable tubers achieved using the fertilizer amendments were 136 – 290% and 69 – 238% higher than the values achieved using control treatment during the first and second cycles, respectively. Soil amendment with BSFCOF produced 18 – 50% higher tubers compared to other chitin-fortified BSFCOF during the second cycle, except BSFCOF+2% chitin and BSFCOF+5% chitin.

### 3.3 Impact of BSFCOF and chitin fortified BSFCOF on nematode population density

The chitin‐fortified BSFCOF amendments significantly reduced the number of cysts per 200 g soil by 32 – 87% and 13 – 76% compared to the control, during the first (χ^2 =^ 55.6, df =7, *P*< 0.001) and second cycles (*F*_(7, 24)_ = 9.87, *P*< 0.001), respectively (**Table 2**). The reduction in the number of cysts increased with increase in the rates of chitin inclusion. Soil amendment with BSFCOF + 5% chitin caused the highest reduction in the number of cysts (75 – 87%) that was significantly (*P*< 0.001) higher than the values achieved using other fertilizer amendments by 50 – 81% and 21 – 72% during the first and second cycle, respectively.

**Table 2:** Effect of chitin-fortified black soldier fly-composted organic fertilizer on potato cyst nematode population parameters.

Treatments	Cycle one					Cycle two				
	Number of cysts/200gsoil	Number of eggs and J2/cyst	Number of eggs and J2/200g-soil	Multiplication rate (*pf/pi*)	Percentage reduction	Number of cysts/200g-soil	Number of eggs/and 2/cyst	Number of eggs and J2/200g-soil	Multiplication rate (*pf/pi*)	Percentage reduction
Control	28.75 ± 4.78a	244.95 ± 20.36a	33.9 ± 3.25a	16.9 ± 1.63a	0	38.5 ± 4.7a	138.0 ± 11.41a	26.1 ± 2.47a	13.0 ± 1.24a	0
BSFCOF	15 ± 3.03bc	156.48 ± 11.81abc	11.2 ± 1.46ab	5.6 ± 0.73b	66.9	29.0 ± 5.46abc	125.5 ± 7.37a	18.1 ± 3.18ab	9.0 ± 1.59ab	30.8
BSFCOF+0.5% chitin	19.5 ± 2.38ab	184.43 ± 2.60ab	18.0 ± 2.33b	9.0 ± 1.1b	50.3	33.5 ± 4.11ab	69.8± 11.03ab	11.9 ± 2.80abc	5.9 ± 1.4abc	54.6
BSFCOF+1% chitin	15.25 ± 2.69bc	128.05 ± 25.19bc	9.6 ± 2.83ab	4.8 ± 1.42bc	71.6	22.3 ± 3.07abc	91.3 ± 11.84bc	10.1 ± 1.62abc	5.1 ± 0.81abc	60.8
BSFCOF+2% chitin	12 ± 3.03bc	120.33 ± 39.98bc	7.9 ± 3.61ab	3.9 ± 1.80bc	76.9	23.8 ± 1.11abcd	41.5 ± 5.81abc	5.0 ± 0.89bcd	2.5 ± 0.44bcd	80.8
BSFCOF+3% chitin	8.75 ± 1.93bc	131.05 ± 20.58bc	5.4 ± 1.34c	2.7 ± 0.67c	84.0	13.3 ± 2.56cd	39.5 ± 17.71cd	2.2 ± 0.87de	1.1 ± 0.44de	91.5
BSFCOF+4% chitin	7.5 ± 1.66bc	90.13 ± 18.58bc	3.8 ± 1.62c	1.9 ± 0.81c	88.0	11.3 ± 2.53d	14.5 ± 7.97d	0.8 ± 0.48e	0.4 ± 0.24e	96.9
BSFCOF+5% chitin	3.75 ± 1.44c	76.1 ± 12.89c	1.2 ± 0.24c	0.6 ± 0.12c	96.4	9.3 ± 1.11d	10.8 ± 8.15d	0.5 ± 0.41e	0.3 ± 0.21e	97.8
Significance level	***	**	***	***		***	***	***	***	

Control, no fertilizer amendment; BSFCOF, black soldier fly-composted organic fertilizer; BSFCOF+0.5% chitin, black soldier fly-composted organic fertilizer amended with 0.5% BSF pupal chitin; BSFCOF+ 1% chitin, black soldier fly-composted organic fertilizer amended with 1% BSF pupal chitin; BSFCOF+ 2% chitin, black soldier fly-composted organic fertilizer amended with 2% BSF pupal chitin; BSFCOF+ 3% chitin = black soldier fly-composted organic fertilizer amended with 3% BSF pupal chitin; BSFCOF+ 4% chitin, black soldier fly-composted organic fertilizer amended with 4% BSF pupal chitin; BSFCOF+ 5%, chitin black soldier fly-composted organic fertilizer amended with 5% BSF pupal chitin. Per column, mean (± standard error) followed by the same letters are not significantly different at p< 0.05. *** P <0.001, ** P <0.01.

It was noted that BSFCOF treatments significantly reduced the number of eggs and J2 per cyst by 25 – 69% and 9.0 – 92% compared to the control during the first (*F*_(7, 24)_ = 6.25, *P<* 0.01) and second cycle (χ^2 =^ 141.8, df =7, *P<* 0.001), respectively (**Table 2**). The highest reduction rate was achieved using BSFCOF + 5% chitin, and this was 16 – 56% and 26 – 91% higher than the values obtained using other BSFCOF and chitin treatments during the first and second cycles, respectively. Likewise, BSFCOF and chitin treatments significantly reduced the number of cyst eggs and J2 per 200 g soil^-1^ by 48 – 96% and 31– 98% compared to unamended soil during the first cycle (χ^2 =^ 141.4, df =7, *P*< 0.001) and second (χ^2 =^ 162.8, df =7, *P*< 0.001) cycles, respectively (**Table 2**). The reduction increased significantly (*P*< 0.001) with an increase in chitin inclusions, and the highest value was achieved using BSFCOF + 5%, which was 69 – 93% and 39 – 97% higher than the values obtained using other fertilizer treatments during in the first and second cycle, respectively.

Soil amendment with BSFCOF and chitin fortified BSFCOF caused significant differences in PCN multiplication rate. BSFCOF and chitin-fortified BSFCOF treatments significantly reduced the reproduction rate by 47– 96% and 31– 98% compared to the unamended soil during the first (χ^2 =^ 141.49, df =7, *P*< 0.001) and second (χ^2 =^ 162.88, df =7, *P*< 0.001) cycles, respectively (**Table 2**). The nematode reproduction rate reduced significantly (*P*< 0.001) with an increase in chitin inclusion. Soil amendment with BSFCOF+5% chitin caused the highest reduction in PCN multiplication rate, that was significantly (*P*< 0.001) higher than the values achieved using other fertilizer amendments by 68 – 93% and 25 – 97% during the first and second cycles, respectively.

### 3.4 Impact of BSFCOF and chitin-fortified BSFCOF on soil chemical properties

The pH of the amended soils was significantly influenced by the fertilizer amendments (first cycle: *F*_(7, 24)_ = 3.91, P< 0.01, second cycle: *F*_(7, 24)_ = 7.36, P< 0.001) (**Table 3**). An increase in the pH of the soil from 7.65 – 8.21 and 7.61– 7.98 indicating a neutral soil pH was observed in the first and second growing cycles respectively. In the first cycle, the fertilizer amendments increased the soil pH by 0.2 – 0.6 log units compared to the unamended soil. Soil amendment with BSFCOF+2% chitin caused the highest increase in soil pH, which was higher than the values achieved with other fertilizer amendments by 0.1– 0.7 log units. In the second cycle, the fertilizer amendments increase the soil pH by 0.02 - 0.4 log units compared to the unamended soil. Soil amendment with BSFCOF + 4% chitin caused the highest increase in soil pH, that was significantly different from other fertilizer amendments by 0.1– 0.4 units.

**Table 3:** Effect of chitin-fortified blacksoldier fly-composted organic fertilizer on selected soil chemical properties.

Experimental cycle	Treatments	pH(H_2_0)	Organic carbon (%)	Ammonium (ppm)	Nitrate (ppm)	Available phosphorus (ppm)	Exchangeable potassium (Cmol kg^−1^)	Exchangeable calcium (Cmol kg^−1^)	Exchangeable magnesium (Cmol kg^−1^)	Exchangeable sodium (Cmol kg^−1^)	Cation exchange capacity (Cmol kg^−1^)
Cycle one	Control	7.65 ± 0.10bc	1.06 ± 0.05c	5.04 ± 0.56b	20.90 ± 3.91a	157.5 ± 5.61b	0.30 ± 0.05a	10.21 ± 0.52ab	1.99 ± 0.1ab	2.14 ± 0.13a	15.57 + 0.78ab
	BSFCOF	7.89 ± 0.06abc	1.71 ± 0.09a	9.24 ± 1.19a	14.32 ± 2.08a	299 ± 19.5a	0.49 ± 0.07a	11.28 ± 0.56a	2.5 ± 0.13a	2.39 ± 0.1a	17.33 ± 0.91a
	BSFCOF+0.5% chitin	7.52 ± 0.03c	1.56 ± 0.37ab	8.04 ± 0.27ab	30.6 ± 8.62a	307.75 ± 21.96a	0.59 ± 0.10a	11.04 ± 0.52a	2.39 ± 0.08b	2.34 ± 0.18a	17.02 ± 0.71a
	BSFCOF+1% chitin	8.06 ± 0.06abc	1. 33 ± 0.12abc	8.86 ± 0.90a	15.65 ± 3.18a	191 ± 2.86b	0.51 ± 0.06a	8.64 ± 0.21b	2.00 ± 0.05ab	2.23 ± 0.05a	13.88 ± 0.34b
	BSFCOF+2% chitin	8.21 ± 0.07a	1.29 ± 0.09abc	7.74 ± 0.66ab	28.3 ± 15.48a	198.75 ± 8.72b	0.50 ± 0.07a	8.77 ± 0.63b	1.93 ± 0.20b	2.24 ± 0.16a	14.12 ± 0.99ab
	BSFCOF+3% chitin	8.13 ± 0.13ab	1.18 ± 0.10bc	6.78 ± 0.83ab	11.86 ± 1.66a	191 ± 5.37b	0.54 ± 0.06a	8.68 ± 0.19b	1.98 ± 0.06ab	2.13 ± 0.03a	13.78 ± 0.27b
	BSFCOF+4% chitin	7.91 ± 0.21abc	1.20 ± 0.04bc	7.74 ± 0.4ab	25.51 ± 13.49a	187 ± 11.69b	0.55 ± 0.05a	8.88 ± 0.43b	1.96 ± 0.09b	2.33 ± 0.14a	14.21 ± 0.72ab
	BSFCOF+5% chitin	8.01± 0.16abc	1.33 ± 0.07abc	9.01 ± 0.79a	14.44 ± 4.68a	191.25 ± 6.91b	0.47 ± 0.02a	9.26 ± 0.27ab	1.98 ± 0.04ab	2.19 ± 0.06a	14.40 ± 0.35ab
	Significance level	**	**	*	ns	***	ns	***	**	ns	**
Cycle two	Control	7.61 ± 0.06bc	1.66 ± 0.05c	5.27 ± 1.70a	4.72 ± 1.65ab	2.35 ± 0.07c	0.87 ± 0.05ab	9.41 ± 0.31b	2.16 ± 0.07b	2.02 ± 0.24b	15.00 + 0.60c
	BSFCOF	7.71 ± 0.07bc	2.34 ± 0.07ab	5.83 ± 0.97a	7.50 ± 1.43ab	56.68 ± 5.78b	0.76 ± 0.09ab	9.40 ± 0.31b	3.00 ± 0.09a	3.44 ± 0.28a	17.23 ± 0.71abc
	BSFCOF+0.5% chitin	7.57 ± 0.03c	2.43 ± 0.25ab	7.72 ± 1.11a	20.0 ± 3.62b	109.47 ± 13.01a	0.97 ± 0.11a	9.16 ± 0.40b	3.40 ± 0.15a	3.43 ± 0.17a	17.65 ± 0.68ab
	BSFCOF+1% chitin	7.59 ± 0.03c	2.48 ± 0.18ab	8.83 ± 1.15a	18.92 ± 6.97b	80.03 ± 21.19ab	0.51 ± 0.06b``	8.95 ± 0.19b	3.02 ± 0.14a	3.02 ± 0.22a	16.27 ± 0.32bc
	BSFCOF+2% chitin	7.63 ± 0.06ab	2.44 ± 0.11ab	7.17 ± 1.74a	11.03 ± 4.30b	89.22 ± 10.64ab	0.50 ± 0.07ab	9.47 ± 0.21b	3.18 ± 0.07a	3.29 ± 0.18a	17.32 ± 0.35abc
	BSFCOF+3% chitin	7.85 ± 0.07ab	2.06 ± 0.12bc	9.22 ± 0.94a	13.20 ± 5.95b	70.47 ± 8.21ab	0.54 ± 0.06b	9.74 ± 0.24ab	3.06 ± 0.07a	3.44 ± 0.25a	17.45 ± 0.51abc
	BSFCOF+4% chitin	7.98 ± 0.04a	2.32 ± 0.06ab	6.35 ± 0.65a	15.16 ± 6.21b	64.53 ± 4.41ab	0.55 ± 0.05b	10.22 ± 0.37ab	3.00 ± 0.10a	3.78 ± 0.15a	18.23 ± 0.65ab
	BSFCOF+5% chitin	7.74 ± 0.04abc	2.77 ± 0.10a	6.96 ± 1.26a	32.62 ± 8.32a	91.97 ± 3.12ab	0.47 ± 0.02ab	10.96 ± 0.11a	3.15 ± 0.05a	3.86 ± 0.18a	19.42 ± 0.28a
	Significance level	***	***	ns	**	***	**	***	***	***	***

Control, no fertilizer amendment; BSFCOF, black soldier fly-composted organic fertilizer; BSFCOF+0.5% chitin, black soldier fly-composted organic fertilizer amended with 0.5% BSF pupal chitin; BSFCOF+ 1% chitin, black soldier fly-composted organic fertilizer amended with 1% BSF pupal chitin; BSFCOF+ 2% chitin, black soldier fly-composted organic fertilizer amended with 2% BSF pupal chitin; BSFCOF+ 3% chitin, black soldier fly-composted organic fertilizer amended with 3% BSF pupal chitin; BSFCOF+ 4% chitin, black soldier fly-composted organic fertilizer amended with 4% BSF pupal chitin; BSFCOF+ 5%, chitin black soldier fly-composted organic fertilizer amended with 5% BSF pupal chitin. Per column, mean (± standard error) followed by the same letters are not significantly different at p< 0.05. ns = non-significant. *** P <0.001, ** P <0.01.

Significant improvements in the soil organic carbon content of the amended soils were observed in the first (*F*_(7, 24)_ = 4.08, *P<* 0.01) and second (*F*_(7, 24)_ = 5.98, *P<* 0.001) cycles of the experiments, respectively. The fertilizer amendments increased the organic carbon content of the soil by 1.1 – 2.3% and 1.7 – 2.8% in the first and second cycles, respectively. In the first growing cycle, the fertilizer amendments increased the soil organic carbon content by 11– 61% compared to the unamended soil. Soil amendment with BSFCOF had the highest organic carbon content, which was significantly different from other fertilizer amendments by 9 – 31%. Compared to the unamended soil, the fertilizer amendments increased the organic carbon content by 24 – 67% in the second cycle. However, soil amendment with BSFCOF + 5% chitin achieved the highest increase in soil organic carbon, which was 10 – 26% higher than the values obtained from other fertilizer amendments.

The BSFCOF and chitin-fortified BSFCOF amendments significantly improved the soil ammonium nitrate (NH^+^_4_-N) concentrations in the first growing cycle (*F*_(7, 24)_ = 3.39, *P*< 0.05) but not in the second cycle (*F*_(7, 24)_ = 1.25, *P* =0.315). The NH^+^_4_-N concentration increased with both applications of BSFCOF singly and with an increase in the percentage of chitin inclusions. The fertilizer amendments increased the soil ammonium nitrate by 2 – 4-fold higher compared to the control. However, soil amendment with BSFCOF had the highest increase in soil ammonium nitrate, which was 0.2 – 2-fold higher than the values obtained from other fertilizer amendments. Significant (*P*< 0.01) increase in the nitrate nitrogen (NO^-^_3_) content of the amended soil was observed in the second cycle (χ^2 =^ 18.608, df =7, *P*< 0.01), but not in the first cycle (*F*_(7, 24)_ = 0.747, *P* =0.635). The frass fertilizer amendments increased the soil NO^-^_3_ by 3 – 28- fold higher compared to the unamended soil. The highest increase in the soil nitrate concentration achieved using BSFCOF + 5% chitin, was 13 – 25-fold higher than the values obtained from other fertilizer amendments.

The available phosphorous concentration of the soil improved significantly (first cycle: χ^2 =^ 152.58, df =7, *P*< 0.001, second cycle*: F*_(7, 24)_ = 9.67, *P*< 0.001) with the application of BSFCOF and chitin fortified BSFCOF amendments. The frass fertilizer amendments increased the available phosphorous concentration of the soil by 30 – 150-fold and 54 –107 fold-higher compared to the unamended soil in the first and second cycles, respectively. In both cycles, soil amendment with BSFCOF + 0.5% chitin achieved the highest increase in the available phosphorous concentration of the soil, which was 9 – 123-fold and 15 – 53-fold higher than the values achieved from other fertilizer amendments in the first and second cycles, respectively.

Significantly improvements in the exchangeable calcium concentration of the amended soil were observed in the first (*F*_(7, 23)_ = 5.87, *P*< 0.001) and second cycle (*F*_(7, 23)_ = 5.26, *P*< 0.001) cycles of the experiments, respectively. In the first cycle, soil amendment with BSFCOF had the highest increase in exchangeable calcium concentration, that was 9% and 2% higher than the values obtained from unamended soil and BSFCOF + 0.5% chitin, respectively. There was a reduction in the exchangeable calcium concentration of the soils amended with chitin compared to the control. In the second growing cycle, there was no significant differences between control treatment and BSFCOF, BSFCOF + 0.5% chitin, BSFCOF + 1% chitin, BSFCOF + 2% chitin and BSFCOF + 3% chitin. However, soil amendment with BSFCOF + 5% chitin had the highest exchangeable calcium concentration that was 7 – 18% higher than the values obtained from other fertilizer amendments.

The exchangeable potassium concentration of the soil differs significantly in the second cycle (*F*_(7, 24)_ = 3.818, *P*< 0.01), but not in the first cycle (*F*_(7, 24)_ = 1.876, *P* = 0.118). There was a reduction in the exchangeable potassium concentration of the soil when compared to the control. Soil amendment with BSFCOF + 0.5% chitin achieved the highest increase in the exchangeable potassium concentration which was 10 – 52% different from Control and other fertilizer amendments. The frass fertilizer amendments increased the exchangeable magnesium concentration of the soil in the first (*F*_(7, 24)_ = 3.92, *P*< 0.01) and second (χ^2 =^ 99.741, df =7, *P*< 0.001) cycles of the experiment, respectively. In the first cycle, soil amendment with BSFCOF had the highest exchangeable magnesium concentration, which was significantly different from other fertilizer amendments by 4 – 23%. In the second cycle, the fertilizer amendments increased the exchangeable magnesium concentration of the soil by 7 – 36%. Soil amendment with BSFCOF + 0.5% chitin achieved the highest exchangeable magnesium concentration, that was different from other fertilizer amendments by 7 – 12%. The exchangeable sodium concentration of the soil was significantly improved in the second cycle (LR χ^2^ = 51.627, df =7*, P*< 0.001) but not significant in the first cycle (*F*_(7, 23)_ = 0.64, *P* = 0.715). The fertilizer amendments increased the exchangeable sodium concentration by 1 – 1.8-fold higher. The highest increase in the exchangeable sodium concentration of the soil achieved using BSFCOF + 5% chitin was 0.1 – 0.8-fold higher than the values achieved using other fertilizer amendments.

The soil cation exchange capacity (CEC) varied significantly due to the fertilizer amendments (first cycle: *F*_(7, 24)_ = 4.30, *P<* 0.01, second cycle: *F*_(7, 24)_ = 6.78, *P*< 0.001). In the first cycle, soil amendment with BSFCOF had the highest CEC, which was 0.3 – 3.6-fold higher than the values obtained from other fertilizer sources. A reduction in the CEC values in soils amended with BSFCOF fortified with 1-5% chitin was observed compared to the control and other fertilizer treatments. In the second cycle, the fertilizer treatments increased the CEC values by 1– 4-fold higher compared to the control. The highest CEC value obtained from soil amendment with BSFCOF + 5% chitin was 1.2 – 3.2-fold higher than the values obtained from other fertilizer amendments.

### 3.5 Multivariate analysis of soil chemical properties, potato growth, yield and nematode population parameters

**Figure 3:**
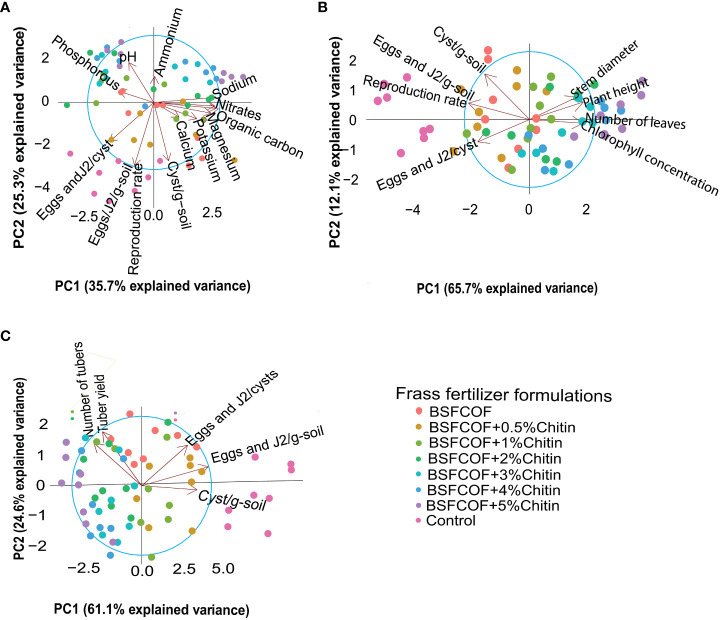
Biplots showing the relatinoship between selected soil chemical properties and nematode population parameters **(A)**, nematode population parameters and pototo growth parameters **(B)**, nematode population parameters and pototo yield parameters **(C)** for the first two principal components (PC1 and PC2). Control, no fertilizer amendment; BSFCOF, black soldier fly-composted organic fertilizer; BSFCOF+0.5% chitin, black soldier fly-composted organic fertilizer amended with 0.5% BSF pupal chitin; BSFCOF+ 1% chitin, black soldier fly-composted organic fertilizer amended with 1% BSF pupal chitin; BSFCOF+ 2% chitin, black soldier fly-composted organic fertilizer amended with 2% BSF pupal chitin; BSFCOF+ 3% chitin, black soldier fly-composted organic fertilizer amended with 3% BSF pupal chitin; BSFCOF+ 4% chitin, black soldier fly-composted organic fertilizer amended with 4% BSF pupal chitin; BSFCOF+ 5%, chitin black soldier fly-composted organic fertilizer amended with 5% BSF pupal chitin.

The BSFCOF and chitin-fortified BSFCOF amendments had a significant impact on the selected soil chemical properties, potato growth, yield, and nematode population parameters, as indicated by the principal component analysis (PCA) results (**Figure 3**). In terms of the relationships between soil chemical properties and nematode population parameters (**Figure 3A**), the first two components of the PCA explained 61% of the total variance, where by, PC1 accounted for 35.7% and PC2 contributed 25.3%. The nematode population parameters exhibited negative correlations with most soil chemical properties especially, soil pH, ammonium nitrate (NH^+^_4_-N), and available phosphorous.

In the case of potato growth and nematode population parameters (**Figure 3B**), the first two components accounted for 77.8% of the total variance; PC1 explained 65.7% of the variance (65.7%), while PC2 accounted for 12.1%. The potato growth parameters correlate negatively with the nematode population parameters especially numbers of eggs and J2 per cyst. Similarly, for yield and nematode population parameters (**Figure 3C**), PC1 and PC2 explained 61.1% and 24.6% of the variance, respectively. Combined, these two components represented 85.7% of the total variance. The number of cysts per g soil and number of eggs and J2/cyst correlates negatively with the yield parameters.

## 4 Discussion

### 4.1 Effect of chitin fortified BSFCOF on potato growth and yield

The present research assessed the benefits of chitin-fortified black soldier fly-composted organic fertilizer on selected soil chemical properties, potato growth and yield, and suppression of potato cyst nematodes. Previous studies investigating the efficacy of BSF-composted organic fertilizer for enhancing soil fertility and crop yield have mainly concentrated on the utilization of composted frass fertilizer alone, without exploring the pesticidal properties of the BSF exuviae which contains chitin (Beesigamukama et al., 2020a, Beesigamukama et al., 2020b; Anyega et al., 2021). Although frass already contains some exuviae (Quilliam et al., 2020), intentional incorporation of chitin-rich exuviae into the frass fertilizer could boost the biopesticide properties of BSFCOF and make it a multipurpose product with the benefits of improving soil health, crop yield, and plant health through suppression of pests and plant pathogens.

The enhanced potato growth and yield achieved using BSFCOF and chitin-fortified BSFCOF during our study have been previously reported on other crops, and could be attributed to the higher nutrient availability, and synchrony for plant growth associated with BSF frass fertilizer (Beesigamukama et al., 2020a, Beesigamukama et al., 2020b; Anyega et al., 2021). Insect frass fertilizer also supplies growth hormones, and beneficial microbes that enhance plant growth and root elongation, leading to higher chlorophyll, number of leaves and stem diameter observed during this study (Barragán-Fonseca et al., 2022; Wantulla et al., 2023b). On the other hand, the higher chlorophyll, leaf growth, stem diameter achieved using chitin-fortified BSFCOF are consistent with previous studies which reported that soil amendment with chitin enhances plant growth by improving the accessibility of nutrients, especially nitrogen (Spiegel et al., 1988b; Debode et al., 2016; Egusa et al., 2019; Li et al., 2020; Poveda, 2021). This is because microbial breakdown of chitin transforms organic nitrogen into an inorganic form, making it accessible for plant absorption (Spiegel et al., 1988b; Roberts and Jones, 2012). Also, the addition of chitin-fortified black soldier fly composted organic fertilizer to the soil could have potentially stimulated the growth and activity of plant growth-promoting rhizobacteria (PGBR), plant growth-promoting fungi (PGBF) and gene expression (Li et al., 2020; Debode et al., 2016; Barragán-Fonseca et al., 2022). Other studies have demonstrated that chitin and chitin-rich organic amendments applied to the soil can stimulate the proliferation of these beneficial microorganism (Cretoiu et al., 2013; Sarathchandra et al., 1996; Radwan et al., 2012).

The improved growth and yield of potato as observed in this study could also be attributed to the increased nematode suppression by the chitin-fortified BSFCOF. Without adequate management practices in place, PCN can reduce potato growth and yield by up to 85% and 75% respectively (Berrahia and Sellami, 2022). This growth and yield reduction can occur even at a low population of 0.1 eggs and J2 per g soil (Maneva and Trifonova, 2015). However, reduction in the population of potato cyst nematodes and improved potato growth and yield due to organic soil amendments as observed in this study have been reported (Renčo and Kováčik, 2015; Ebrahimi et al., 2016; Renčo et al., 2016; Renčo et al., 2011). The results obtained in this study are consistent with the findings of Boukhlifi et al. (2018), who reported an increase in the growth and yield of wheat and potato growth parameters with the application of raw chitin from shrimp waste. Furthermore, Debode et al. (2016), Lee et al. (2005), Kim et al. (2005), and Ait Barka et al. (2004) reported significant improvements in the growth of tomato, grapevine, lettuce, sweet basil, and improvements in soybean sprouts due to chitin and chitosan applications. It is important to note that crops respond differently to chitin source and rates of application. For instance, Mian et al. (1982) observed that soil amendments with crustacean chitin flakes at a rate above 2% reduced the seed germination of crookneck squash, while application rates above 1% reduced shoot weight. On the contrary, Muymas et al. (2014) reported significant improvements in growth, yield and postharvest quality of lettuce grown in soil amended with 5% shrimp shell chitin powder and 20% of fermented chitinous material.

We did not observe any negative effect of chitin on both plant growth and yield in the present study. However, the potato yields obtained using the unfortified BSFCOF were not significantly different from the yield achieved using the chitin-fortified BSFCOF even at the highest rate of 5% inclusion in the first cycle, but was significantly different from other fertilizer amendments in the second cycle except BSFCOF+2% chitin and BSFCOF+5% chitin. The additional advantages of improved plant growth and increased nematode suppression achieved using chitin inclusions makes the chitin-fortified BSFCOF a multipurpose organic fertilizer and a better alternative to unfortified BSFCOF. The present study did not investigate the impact of BSFCOF formulations on growth-promoting rhizobacteria and other beneficial organisms; therefore, future studies are warranted to demonstrate the benefits of chitin-fortified BSFCOF on beneficial soil organisms, enzymes and growth hormones.

### 4.2 Effect of chitin fortified BSFCOF on nematode population density and soil chemical properties

The significant reduction in the number of cysts/200 g soil^-1^, number of eggs and J2/cyst, the number of eggs per g^-1^ soil, and nematode multiplication rate achieved in this study indicate the nematicidal activity of BSFCOF and chitin-fortified BSFCOF and their potential for use as a biorational pesticide. The improved nematode suppression could be attributed to the high nitrogen content and reduced C:N ratio of the chitin-fortified black soldier fly composted organic fertilizer and the chitin present in the pupal exuviae (Rodriguez-Kabana et al., 1987; Renčo et al., 2010). Furthermore, the release of nitrogenous compounds, organic acids, and other by-products of organic matter decomposition are some of the key factors regulating the population of plant-parasitic nematodes (Oka and Yermiyahu, 2002; Oka, 2010; Thoden et al., 2011; Rodríguez-Kábana, 1986; Mian et al., 1982). Organic materials with low C:N ratios such as BSFCOF and chitin-fortified BSFCOF used in the present study have been shown to be more effective in controlling plant-parasitic nematodes (Rodriguez-Kabana et al., 1987; Nico et al., 2004; Renčo et al., 2010) due to faster decomposition, high ammonium release, and the presence of other toxins (Rodriguez-Kabana et al., 1987; Oka and Yermiyahu, 2002; Rodriguez-Kabana, 1984; Mian et al., 1982; Rodriguez-Kabana et al., 1987). The negative correlation between nematode population parameters and soil pH, Ammonium nitrogen and other soil chemical properties as shown by the principal component analysis confirms these reports. Also, the rise in soil pH might have enhanced PCN suppression by stimulating ammonium release, leading to improved nematicidal effects (Oka et al., 2006a, Oka et al., 2006b; Oka et al., 2007; Oka, 2010; Zasada, 2005).

The observed suppressive effect of the chitin-fortified BSFCOF on the potato cyst nematodes could also be attributed to the activation of some chitinolytic microorganisms that prey on the nematode eggs and egg sacs, leading to a decrease in their capacity to reproduce and invade the potato roots (Spiegel et al., 1987; Rodriguez-Kabana et al., 1986). In a recent study, Nyang’au et al. (2023) reported that certain fungal species isolated from Kenyan population of PCN reduced cyst egg viability by up to 41% and hatchability by 50% under *in vitro* conditions. The reduction in number of cysts per g soil, number of eggs and J2 per cyst, number of eggs per g^-1^ soil and multiplication rate confirms the nematicidal potentials of black soldier fly composted organic fertilizer and the chitin-rich exuviae as a biorational fertilizer in improving potato productivity. Also, it is pertinent to note that this reduction increased with an increase in the pupal exuviae chitin inclusion, providing justification for fortification of BSF-composted organic fertilizer with pupal chitin. Our findings have shown that a chitin amendment rate of 5% is sufficient for boosting potato yield and suppression PCN. The reduction in number of eggs and J2 per cyst achieved in the study is higher than the value reported by Renčo et al. (2007) while using organic materials of varying sources. Also, our study demonstrated the potential of chitin-fortified BSFCOF to reduce PCN reproduction rate by 98%, which is higher than the values (82 – 87%) achieved by Ebrahimi et al. (2016) while using pig slurry and wood chips for nematode control. These findings highlight the superiority of BSF pupal chitin and frass fertilizer in suppressing nematode pests compared to other organic amendments, and are consistent with previous studies that have reported the efficacy of compost, chitin, and chitin-rich organic soil amendments in suppressing nematodes (Renčo et al., 2010; Renčo and Kováčik, 2015; Ebrahimi et al., 2016; Renčo et al., 2016).

The significant improvements in the soil chemical properties observed during the study demonstrate the capability of the BSCOF and pupal exuviae chitin to boost soil fertility and create a favorable environment for crop performance. The increase in soil pH observed in this study has been previously reported (Beesigamukama et al., 2021; Menino et al., 2021). Our findings indicates the ability of the BSCOF and chitin-fortified BSFCOF in alleviating soil acidity which is a key challenge to crop production in sub-Saharan Africa (Tully et al., 2015; Montanarella et al., 2016). The slight rise in soil organic carbon content observed in this study could be linked to the stability of carbon in soils amended with frass, which has been previously reported (Gebremikael et al., 2022), and highlights the benefit of BSFCOF in enhancing soil carbon sequestration and replenishing soil organic matter (Axelrod et al., 2021; Guo et al., 2021; Menino et al., 2021; Watson et al., 2021; Zhang et al., 2017). Nevertheless, mid or long-term studies are recommended for thorough investigation of changes in soil organic carbon due to BSF-composted organic fertilizer and pupal chitin amendment. Previous studies (Beesigamukama et al., 2021; Gebremikael et al., 2022) have reported an increase in the concentration of ammonium in frass amended soils, which was also observed in this study. Our study showed a higher concentration of NO_3_-N compared to NH_4_-N in the amended soils and this indicates the role of BSF-composted organic fertilizer and pupal chitin amendment in improving the nitrification process. The inclusion of pupa exuviae chitin with a low C:N ratio of 7.6 in the BSFCOF might have increased the rate of mineralization, resulting in faster release of N and reduced N immobilization by microbes. Conversely, our results contradict those of Beesigamukama et al. (2021) who reported a higher NH_4_-N in BSFCOF-amended soil, probably because their studies did not involve BSF pupal exuviae.

The significant increase in the concentration of available phosphorus in soils amended with chitin-fortified BSFCOF is consistent with previous findings (Agustiyani et al., 2021; Beesigamukama et al., 2021; Guo et al., 2021; Menino et al., 2021) and demonstrates the benefit of insect frass fertilizer in addressing the challenge of phosphorous fixation in the highly-weathered soils of Africa and tropical regions (Tully et al., 2015). Organic soil amendments such as insect composted frass fertilizer have been reported to increase phosphorous availability through reduction in soil pH and release of organic acids, chelation of cations responsible for phosphorous fixation such as calcium, magnesium, iron and aluminium, and by stimulating the activities of phosphate solubilizing bacteria (Ghosh et al., 2021; Adnan et al., 2022).

The enhancement of exchangeable cations (calcium, magnesium, potassium and sodium) and cation exchange capacity (CEC) observed during the study could be largely attributed to improved soil buffer capacity and increase in the net negative charges introduced by organic amendments in the exchange complex. Cation exchange capacity is critical in enhancing soil nutrient retention and availability for plant uptake. The increased soil buffer capacity could have also increased the availability of basic cations as previously reported by Mucheru-Muna et al. (2007) while using organic soil amendments. Our findings align with those of Menino et al. (2021) and Guo et al. (2021) and support the potential of chitin-fortified BSFCOF as an effective organic soil amendment for improving soil fertility. Therefore, the multipurpose and regenerative BSFCOF and chitin-fortified BSFCOF products could be considered as partial or complete substitutes to the low-quality, ineffective, and inaccessible organic fertilizers and pesticides as well as the scarce and costly synthetic fertilizers and pesticides used in most cropping systems across sub-Saharan African and other tropical regions. The commercialization of BSFCOF and allied products is already taking shape globally, with over 2300 enterprises established in Africa (Tanga and Kababu, 2023). Kenya is among the leaders of insect farming enterprises in Africa; BSFCOF is traded in more than 1400 agro-input shops in the country at a cost of 16 USD per 50 kg bag (Tanga and Kababu, 2023).

## 5 Conclusion

This study has demonstrated the potential of BSFCOF and chitin-fortified BSFCOF as multipurpose organic amendments for improving soil fertility, potato growth and yield, and suppressing potato cyst nematodes. The higher potato yield, and nematode suppression in soil as well as reduced reproduction rate achieved during the study indicate multipurpose nature of BSFCOF and chitin-fortified in boosting crop productivity while suppressing soil-borne pests. For effective management of PCN and enhancement of potato yield, it is recommended that BSFCOF is combined with 5% BSF pupal exuviae chitin. Widespread adoption of this chitin-fortified BSFCOF among smallholder farmers could reduce their reliance on costly inorganic fertilizers and commercial nematicides, which also have negative environmental implications. Field studies are warranted to validate the findings of this study under open field conditions, compare the performance of chitin-fortified BSFCOF with nematicides and commercial fertilizers in different agroecological zones, and assess the economic value of using chitin-fortified BSFCOF for crop production. Further investigations should be directed towards understanding the mechanisms of nematode suppression associated with BSFCOF and chitin-fortified BSFCOF amendments, and the diversity, abundance and functional roles of microbiome in soils amended with chitin-fortified BSFCOF.

## Data availability statement

The original contributions presented in the study are included in the article/Supplementary Material. Further inquiries can be directed to the corresponding authors.

## Author contributions

EA: Conceptualization, Data curation, Formal Analysis, Investigation, Methodology, Software, Validation, Writing – original draft, Writing – review & editing. DB: Conceptualization, Data curation, Formal Analysis, Investigation, Methodology, Software, Validation, Writing – original draft, Writing – review & editing, Supervision, Visualization. BM: Conceptualization, Investigation, Supervision, Writing – original draft, Writing – review & editing. NK: Conceptualization, Investigation, Supervision, Writing – original draft, Writing – review & editing, Data curation, Formal Analysis, Methodology, Software, Visualization. SH: Conceptualization, Formal Analysis, Investigation, Methodology, Supervision, Writing – original draft, Writing – review & editing, Project administration, Validation. XC: Investigation, Supervision, Writing – original draft, Writing – review & editing, Resources. SS: Resources, Supervision, Writing – original draft, Writing – review & editing, Project administration. SK: Project administration, Resources, Writing – original draft, Writing – review & editing, Funding acquisition. CT: Funding acquisition, Project administration, Resources, Writing – original draft, Writing – review & editing, Conceptualization, Data curation, Formal Analysis, Investigation, Methodology, Software, Supervision, Validation, Visualization.
